# Inflammatory role of microglia in brain injury caused by subarachnoid hemorrhage

**DOI:** 10.3389/fncel.2022.956185

**Published:** 2022-12-06

**Authors:** Xiao-Yi Wang, Fan Wu, Ren-Ya Zhan, Heng-Jun Zhou

**Affiliations:** Department of Neurosurgery, The First Affiliated Hospital, College of Medicine, Zhejiang University, Hangzhou, Zhejiang, China

**Keywords:** early brain injury, inflammation, microglia, neuroinflammation, polarization, role, subarachnoid hemorrhage, single-cell analysis

## Abstract

Early brain injury is a series of pathophysiological changes and direct damage of brain tissue within 72 h after subarachnoid hemorrhage before cerebral vasospasm occurs. Early brain injury is a key factor affecting the prognosis of subarachnoid hemorrhage, and its possible pathological mechanisms include oxidative stress, cell apoptosis, autophagy, and immune inflammation. Microglia are important immune cells of the central nervous system. Microglia play a dual role in protection and injury. Microglia are involved in the occurrence of brain edema, the processes of neuronal apoptosis, and the blood–brain barrier disruption after subarachnoid hemorrhage (SAH) through the signaling pathways mediated by receptors such as Toll-like receptor 4 (TLR4), calcium-sensing receptor (CaSR), and triggering receptor expressed on myeloid cells-1 (TREM-1), which secrete pro-inflammatory cytokines such as interleukins and tumor necrosis factor α. Conversely, they exert their anti-inflammatory and protective effects by expressing substances such as neuroglobin and heme oxygenase-1. This article reviews the latest developments in single-cell transcriptomics for microglia in early brain injury after subarachnoid hemorrhage and its inflammatory role.

## Introduction

Subarachnoid hemorrhage (SAH) is a subtype of stroke with high mortality and poor prognosis, accounting for 3–5% of strokes (Bian et al., 2012; Muehlschlegel, 2018). SAH is caused by blood flowing into the subarachnoid space after the rupture of the intracranial vasculature. Intracranial aneurysm rupture is the most common cause of SAH.

**FIGURE 1:**
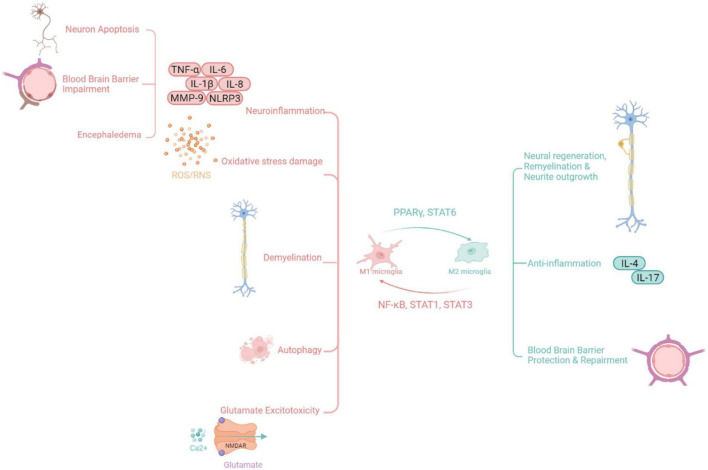
Functions of microglia [Possible pathological mechanisms of M1 microglia after subarachnoid hemorrhage include oxidative stress, neuron apoptosis, autophagy, and immune inflammation. Microglia are also involved in the occurrence of brain edema, the processes of neuronal apoptosis, and the blood–brain barrier (BBB) disruption after subarachnoid hemorrhage (SAH) through inflammation response. Conversely, M2 microglia exert anti-inflammatory and protective effects].

A defining feature of SAH is cerebral vasospasm; however, the treatment of cerebral vasospasm does not improve the outcome of patients. Therefore, cerebral vasospasm may not be the only cause of SAH (Bian et al., 2012). Recent studies have found that early brain injury (EBI) after SAH is important for patient prognosis. EBI refers to direct brain injury and secondary pathophysiological changes within 72 h after SAH before cerebral vasospasm occurs (Rass and Helbok, 2019). As important immune cells of the central nervous system, microglia undergo polarization, secrete pro-inflammatory factors, and recruit immune cells to participate in the neuroinflammatory response in EBI after SAH. Microglia also lead to cerebral edema and destruction of the blood–brain barrier (BBB). Microglia play an important role in hematoma evacuation and brain tissue repair (Coulibaly and Provencio, 2020; Solár et al., 2022). In the inflammatory response, the dual role of microglia often coexists, but microglia mainly play a role in injury in the early stage and a protective role in the late stage of EBI (Chen and Wong, 2021). This article reviews the process of M1/M2 microglial polarization in EBI after SAH and its dual mechanism of action (Figure 1).

## Early brain injury after subarachnoid hemorrhage

Epidemiological studies have shown that about half of patients with SAH die within 30 days after onset, and about two-thirds of them die within 24–72 h (Geraghty et al., 2019). Neuronal degeneration and apoptosis can be observed in the brain within 10 min after SAH (Solár et al., 2022). Simultaneously, in EBI, microglia also show morphological signs of activation, leading to neuronal necrosis and the release of inflammatory factors (Coulibaly and Provencio, 2020).

The etiology of EBI after SAH involves a series of pathophysiological mechanisms triggered by the initial hemorrhage, which makes the brain prone to secondary injury (Van Lieshout et al., 2018). The underlying mechanisms of EBI include increased intracranial pressure, decreased cerebral blood perfusion, cerebral edema, neuronal dysfunction, and inflammatory response (Rass and Helbok, 2019). Extravasation of the subarachnoid blood may cause a sudden increase in intracranial pressure, which causes a decrease in cerebral perfusion pressure and impaired autoregulation; in extreme cases, transient and persistent ischemia may occur (Coulibaly and Provencio, 2020). Neuronal cell death and endothelial injury lead to cytotoxic edema and disruption of the BBB, which exacerbate the development of vasogenic edema.

Neuroinflammation plays an important role in the early stages of SAH development, leading to neuronal apoptosis, disruption of BBB, and brain edema, which is characterized by the activation of microglia, infiltration of inflammatory cells, and production of cytokines (Rass and Helbok, 2019). Non-steroidal anti-inflammatory drugs, thromboxane synthase inhibitors, steroids, and immunosuppressive therapies have been tested to treat neuroinflammation after SAH. However, none of these therapies are recommended as routine treatments (Rass and Helbok, 2019). An in-depth understanding of the role of neuroinflammation in EBI after SAH can better provide novel therapeutic strategies for SAH.

## Biological characteristics of microglia

Microglia are tissue-specific macrophages of the nervous system that are derived from primitive yolk sac medullary progenitors and extensively colonize the central nervous system during metaphase embryonic development. Microglia are important mediators of neuroinflammation (van Dijk et al., 2016). The typical morphology of microglia is highly branched, with many short and thin processes at rest. These processes provide a large contact area and extend into the surrounding environment, placing themselves in a good position to facilitate the sensing and monitoring of changes in the local environment. When the central nervous system is injured, microglia are rapidly activated and undergo morphological changes, including enlarging the cell size, shortening the processes, and eventual disappearance of processes. Microglia eventually become amoeboid (Zheng and Wong, 2017).

**FIGURE 2:**
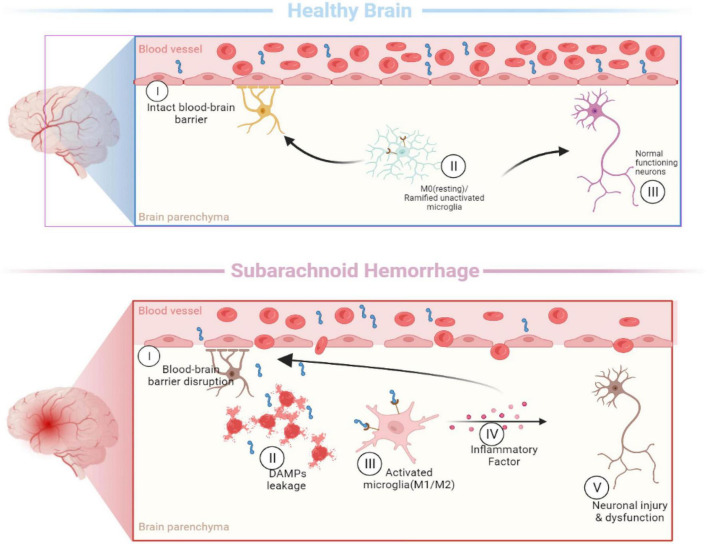
Activation of microglia [In the healthy brain, the typical morphology of M0 microglia is highly branched, with many short and thin processes. When the subarachnoid hemorrhage occurs, microglia are rapidly activated and undergo morphological changes (M1/M2), including enlarging the cell size, shortening the processes, and eventual disappearance of processes].

In the past, microglia were simply classified as “M0” (resting), “M1” (pro-inflammatory), and “M2” (anti-inflammatory) (Xu et al., 2017; Wei et al., 2021) based on their morphological characteristic and functional differences, which cannot explain the diversity of myeloid subpopulations in the diseased brain (Figure 2). These simple classification schemes may complicate this issue by bringing heterogeneous microglia together. Identifying and molecularly describing distinct microglia will help us determine whether microglia exhibit different characteristics depending on the type of injury and disease (Hammond et al., 2019). The development of single-cell sequencing in recent years has greatly advanced the knowledge and understanding of microglial heterogeneity. On the premise of ensuring high-throughput data detection, single-cell sequencing can conduct unbiased and objective detection of the whole gene of a single cell in a cell population and conduct individualized research (Ochocka and Kaminska, 2021).

Different microglia transcriptional states have been found in the central nervous system. Microglia-repressed expression of P2ry12 and Cx3cr1 was identified in a mouse model of multiple sclerosis, and these cells were named injury-responsive microglia (IRM) (Hammond et al., 2019). In addition, a subpopulation of microglia, called disease-associated microglia (DAM) (Keren-Shaul et al., 2017), was found in mouse models of Alzheimer’s disease. And axon tract-associated microglia (ATM) were observed in the developing mouse brain (Hammond et al., 2019). By performing a single-cell transcription analysis of post-SAH microglia, Chen et al. (2022) named the microglia cluster obtained from the SAH model as the SAH microglia cluster (SMG-C). In the SAH model, three distinct microglia subsets of SMG-C5, SMG-C6, and SMG-C7 were observed. SMG-C5 was characterized by the expression of marker genes, including Spp1, Lpl, Ctsb, Lgals1, Apoe, etc. According to previous reports, DAM, ATM, IRM, and SAH-associated microglia share the expression of a core set of genes, including Spp1, Lpl, and Apoe (Hammond et al., 2019). After SAH, the expression levels of the genes Birc5, Mki67, and Fabp5 in SMG-C7 and the metabolically active genes Mif and Ms4a6c in SMG-C5 were upregulated, but not in normal microglia. The differences between the SMG and other microglia suggest that microglia have distinct activation patterns in SAH (Keren-Shaul et al., 2017). The research also found that SMG-C5, SMG-C6, and SMG-C7 had common inflammatory pathway connections, which may affect the occurrence and progression of neuroinflammation after SAH (Chen et al., 2022).

## The role of microglia in early brain injury

### Microglia mediate neuronal apoptosis

**FIGURE 3:**
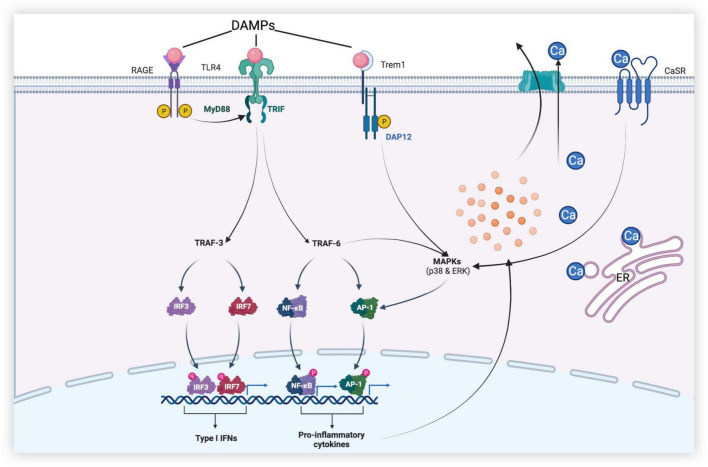
Dural roles of microglia-associated receptors in early brain injury (EBI) after subarachnoid hemorrhage. [After subarachnoid hemorrhage (SAH), danger-associated molecular patterns (DAMPs) activate receptors such as Toll-like receptor 4 (TLR4), receptors for advanced glycation end (RAGE), and triggering receptor expressed on myeloid cells-1 (TREM-1) of microglia. TLR4 produces a series of pro-inflammatory mediators through MyD88-dependent and Toll receptor-associated activator of interferon (TRIF)-dependent pathways, leading to neuronal apoptosis, disruption of the blood–brain barrier (BBB), and brain edema, etc., while promoting the release of type I IFN to play anti-inflammatory and anti-apoptotic roles. In addition, calcium-sensing receptor (CaSR) and TREM-1 promote mitogen-activated protein kinase (MAPK) expression and initiate downstream inflammatory pathways, thereby aggravating brain injury].

In SAH, Toll-like receptor 4 (TLR4) expressed by microglia is activated by ligands such as high-mobility group box protein 1 (HMGB1), heme, and methemoglobin and mediates a series of downstream signaling pathways through adaptor proteins (Figure 3) (Okada et al., 2019). This pathway produces pro-inflammatory cytokines and oxidative metabolites, leading to neuronal apoptosis and tissue damage. TLR4, a member of the Toll-like receptor family, is a pattern recognition receptor that can recognize different pathogen-associated molecular patterns (PAMPs) produced by pathogenic microorganisms *in vitro*, such as lipopolysaccharides (Lehnardt et al., 2003), and danger-associated molecular patterns (DAMPs) released by various injured cells *in vivo*. DAMPs include heme, hemoglobin, and HMGB1 (Xiong et al., 2016). Upon stimulation by these molecules, microglia can initiate an inflammatory cascade to produce the corresponding pro-inflammatory cytokines, such as tumor necrosis factor-α (TNF-α), interleukin-1β (IL-1β), and IL-12. In addition, M1 microglia also present antigens and promote the production of cytotoxic substances by expressing high levels of inducible nitric oxide synthase (iNOS) (Xiong et al., 2016; Pan et al., 2020).

Inflammatory cytokines aggravate brain injury after SAH by triggering apoptotic pathways, interfering with the relaxation and contraction of blood vessels, and recruiting peripheral immune cells by upregulating intercellular adhesion molecules (Geraghty et al., 2019). TLR4 polarized by stimulation can promote the activation of nuclear factor-κB (NF-κB) and the production of downstream cytokines and mediators, such as TNF-α, interleukins (IL-1β, IL-6, IL-8, and IL-12), intercellular adhesion molecule-1, monocyte chemoattractant protein-1, cyclooxygenase, and reactive oxygen species, through the myeloid differentiation factor 88 (MyD88)-dependent pathway (Kawakita et al., 2017; Zheng and Wong, 2017). NF-κB is a key transcription factor associated with M1 microglial activation, and it has been shown that the inhibition of the NF-κB p65/p50 subunit inhibits the transcription of inflammatory genes and promotes microglial conversion to the M2 phenotype (Zhang et al., 2013). In the EBI of SAH, the MyD88-dependent pathway can also activate the pathway mediated by mitogen-activated protein kinase (MAPK) and promote neuronal necrosis and apoptosis by producing factors such as IL-1 and proteases. Under physiological conditions, TNF-α, IL-1, and IL-6 expressed by microglia play a positive role in neuronal synaptic plasticity and memory, but high concentrations of these cytokines may cause damage to neurons and lead to neuronal death. For example, TNF-α overexpression can also activate microglia to exacerbate neuronal apoptosis and brain injury after SAH (Zhang et al., 2016). Although the mechanism of microglial activation by TNF-α has been previously reported, strategies to effectively target it remain unclear (Brás et al., 2020).

Receptors for advanced glycation end (RAGE) products expressed in neurons and microglia, which are members of the immunoglobulin superfamily, can interact with pro-inflammation ligands like HMGB1, the S100 protein family, amyloid β, and macrophage antigen complex-1 to promote the polarization of M1 microglia and the release of inflammatory factors such as TNF-α and IL-6 (Ieong et al., 2018). These inflammatory factors can lead to neuronal apoptosis. As a central signaling molecule in the innate immune system, RAGE play an essential role in initiating and maintaining the inflammatory response (Li et al., 2014). Aberrant upregulation and activation of RAGE have been implicated in various inflammation-related diseases, including diabetes, traumatic brain injury, and ischemic stroke. In the SAH mouse model, RAGE mRNA was significantly increased at 6 h and peaked at 12 h after SAH. In experimental SAH rat models, RAGE are overexpressed in the cerebral cortex near the clots in the subarachnoid space and are mainly found in neurons and microglia. The expression of NF-κB p65 was also significantly increased, demonstrating that RAGE may play a role in pro-inflammation through NF-κB in the early stage of SAH (Li et al., 2014).

### Microglia mediate the disruption of blood–brain barrier and brain edema

The BBB is an important physiological barrier in the human body. When damaged, inflammatory substances and plasma components are more likely to enter the brain parenchyma and further aggravate neuroinflammation and brain injury, causing brain dysfunction. Global brain edema and increased BBB permeability during the acute phase of aneurysmal SAH are associated with adverse outcomes (Kanamaru and Suzuki, 2019). After SAH, MAPK, related to the TLR4/MyD88 signaling pathway, is activated by ligands such as extracellular signal-regulated kinase (ERK), p38, and c-Jun N-terminal kinase (JNK), which further increase the release of cytokines such as matrix metalloproteinases (MMPs), proteases, and interferons (IFNs) (Okada and Suzuki, 2017). The BBB is mainly composed of chemical and structural barriers formed by endothelial tight junction-associated proteins, such as occluding and ZO-1. It has been repeatedly reported that MMP-9 induced by TLR4-mediated signaling pathways can cause BBB disruption after SAH by degrading extracellular matrix proteins and ZO-1 (Nishikawa et al., 2018; Okada et al., 2019). Additionally, periostin, IL-6, and tenascin-C can trigger a cascade leading to BBB disruption by upregulating and activating MMP-9. Periostin and tenascin-C are matricellular proteins that directly interact with each other, causing BBB disruption. MAPKs exist both upstream and downstream of periostin, tenascin-C, and IL-6; activated MAPKs can induce their production, which activates MAPKs, forming a positive feedback mechanism (Okada et al., 2019).

Activation of the calcium-sensing receptor (CaSR) can lead to neurological deterioration, brain edema, and neurodegeneration. CaSR is widely expressed in neurons, astrocytes, and microglia. CaSR agonists promote the phosphorylation of calcium-calmodulin-dependent protein kinase type 2 (CaMKII) and the expression of NLRP3 inflammasome, cleaved caspase-1, and IL-1β, leading to neurological deterioration and aggravation of brain edema (Moitrayee et al., 2020; Wang et al., 2020). The activation of the NLRP3 inflammasome mediates the activation of caspase-1 and promotes the secretion of pro-inflammatory factors, such as IL-1β and IL-18. The use of IL-1β inhibitors reduces the expression of MMP-9 and attenuates BBB injury (Dodd et al., 2021). The NLRP3 inflammasome can also aggravate BBB injury by participating in vascular endothelial cell apoptosis. Therefore, the degree of neurological dysfunction, brain edema, and disruption of the BBB after SAH can be improved by inhibiting the activation of CaSR and the generation of the NLRP3 inflammasome (Li et al., 2016; Luo et al., 2019). Although studies have shown that CaSR causes neurological damage and toxic effects in Alzheimer’s disease and cerebral ischemia (Zhang et al., 2019), studies on SAH are scarce. Therefore, whether CaSR and CaSR-related pathways are effective therapeutic targets requires further investigation.

Moreover, it has been shown that triggering receptors expressed on myeloid cells-1 (TREM-1) on the surface of microglia are increased in the cerebrospinal fluid of patients in the early stage of SAH and can aggravate the disruption of the BBB, neurological deficits, and microglial anxiety by degrading tight junction protein-1 and activating MAPK/MMP-9 and NLRP3 inflammasome (Sun et al., 2019; Xu et al., 2021). It has previously been shown that TREM-1 plays an important role in the inflammatory response caused by ischemic stroke and myocardial infarction. However, whether TREM-1 can regulate the neuroinflammatory response after SAH and its regulatory mechanism remain to be studied largely, which is expected to provide a new therapeutic approach for EBI.

### Neuroprotective effects of microglia

On the first day after SAH, the expression of M1 microglia-related products, such as IL-6 and TNF-α, is significantly upregulated in the brain tissue. On the third day after SAH, the expression of M1 microglia begins to decrease, while that of M2 microglia begins to increase (Chen and Wong, 2021). M2 microglia play a role in allergic reactions, parasite clearance, inflammation inhibition, tissue remodeling, and immune regulation (Orihuela et al., 2016). In SAH, the function of M2 microglia is mainly to promote the repair of injury by inhibiting inflammation, and M2 microglia mainly appear in the late stage of injury in EBI (Hu et al., 2015).

During EBI, microglia activation promotes the formation of a series of neuroprotective proteins, such as neuroglobin and heme oxygenase-1. Neuroprotective proteins protect neurons by reducing oxidative stress. The neuroprotective effect of HO-1 has been demonstrated in a study in which HO-1 expression in microglia was knocked out. At the same time, microglia also upregulate the expression of metabotropic glutamate receptor 5 and reduce the production of pro-inflammatory cytokines such as IL-1β, IL-6, and TNF-α (Zhang et al., 2015), which further reduces neuronal apoptosis and the degree of brain edema (van Dijk et al., 2016). Additionally, activated microglia after SAH also promote the expression of IL-4 and IL-17 (Galea et al., 2018) and play a role in engulfing cell debris and hematoma evacuation through scavenger receptors (Kim et al., 2022).

Toll-like receptor 4 not only plays a role in brain injury but also protects brain tissue through its anti-inflammatory effects. In addition to the MyD88-dependent signaling pathway mentioned above, TLR4 can also send signals through the Toll receptor-associated activator of interferon (TRIF)-dependent cascade, initiating the activation of transcription factors to regulate the gene expression of pro-inflammatory cytokines and coordinate the maximal inflammatory response (Häcker et al., 2006). The TRIF signaling pathway recruits adaptor molecules mediated by TNF receptor-associated factor 3 (TRAF3) and TRAF6 (Häcker et al., 2006). TRIF-dependent signaling pathway mediated by TRAF6 generates inflammatory cytokines through the same cascade as the MyD88-dependent signaling pathway. We refer to the NF-κB activation that depends on TRIF as “late” NF-κB activation, while “early” NF-κB activation depends on the MyD88 pathway. Coordinating “early” and “late” activation is a function unique to TLR4. However, in contrast to TRAF6, the TRAF3-mediated TRIF signaling pathway can induce the production of IFN-β, which plays anti-inflammatory and anti-apoptotic roles. IFN-β also regulates the production of other IFN-Is, thereby further suppressing the inflammatory response after EBI (Jiang et al., 2020).

## Conclusion

Subarachnoid hemorrhage is a cerebrovascular disease with high mortality and high disability rates; effectively treating it based on its complex injury mechanism has always been a research focus. Microglia play an important role in BBB damage, brain edema, and neuronal degeneration during EBI. The inflammatory pathways mediated by TLR4 have been extensively studied, while other receptor-related pathways, such as CaSR and TREM-1, have been less studied. The nerve injury and toxic effects of CaSR and TREM-1 have been demonstrated in other central nervous system diseases, but little research has been conducted on EBI after SAH. The mechanism of their activation and the related signaling pathways may provide important therapeutic targets for SAH in the future.

Identification of transcriptional signatures can provide a better understanding of the role of microglia in resting and disease states. Although single-cell technology can successfully identify various specific cell subtypes exhibited by microglia in different developmental stages and pathological states, the roles and interrelationships of these different subtypes have not been thoroughly studied. Additionally, there are still few studies on SAH models, and future studies on SAH models may provide key information for understanding SAH-related pathology and may develop effective treatments for SAH in the future.

## Author contributions

X-YW and H-JZ formulated the idea and drafted the manuscript. FW revised the draft and drew the figures. R-YZ and H-JZ critically revised the draft. All authors contributed to the article and approved the submitted version.
